# Single Nucleotide Polymorphisms in *HSP17.8* and Their Association with Agronomic Traits in Barley

**DOI:** 10.1371/journal.pone.0056816

**Published:** 2013-02-13

**Authors:** Yanshi Xia, Ronghua Li, Zhengxiang Ning, Guihua Bai, Kadambot H. M. Siddique, Guijun Yan, Michael Baum, Rajeev K. Varshney, Peiguo Guo

**Affiliations:** 1 International Crop Research Center for Stress Resistance, College of Life Sciences, Guangzhou University, Guangzhou, China; 2 College of Light Industry and Food Science, South China University of Technology, Guangzhou, China; 3 Hard Winter Wheat Genetics Research Unit, United States Department of Agriculture - Agricultural Research Service, Manhattan, Kansas, United States of America; 4 The Institute of Agriculture, The University of Western Australia, Crawley, Perth, Australia; 5 International Center for Agricultural Research in the Dry Areas, Aleppo, Syria; 6 International Crops Research Institute for the Semi-Arid Tropics, Patancheru, Greater Hyderabad, India; Ecole Normale Superieure, France

## Abstract

Small heat shock protein 17.8 (HSP17.8) is produced abundantly in plant cells under heat and other stress conditions and may play an important role in plant tolerance to stress environments. However, *HSP17.8* may be differentially expressed in different accessions of a crop species exposed to identical stress conditions. The ability of different genotypes to adapt to various stress conditions resides in their genetic diversity. Allelic variations are the most common forms of genetic variation in natural populations. In this study, single nucleotide polymorphisms (SNPs) of the *HSP17.8* gene were investigated across 210 barley accessions collected from 30 countries using EcoTILLING technology. Eleven SNPs including 10 from the coding region of *HSP17.8* were detected, which form nine distinguishable haplotypes in the barley collection. Among the 10 SNPs in the coding region, six are missense mutations and four are synonymous nucleotide changes. Five of the six missense changes are predicted to be deleterious to *HSP17.8* function. The accessions from Middle East Asia showed the higher nucleotide diversity of *HSP17.8* than those from other regions and wild barley (*H. spontaneum*) accessions exhibited greater diversity than the cultivated barley (*H. vulgare*) accessions. Four SNPs in *HSP17.8* were found associated with at least one of the agronomic traits evaluated except for spike length, namely number of grains per spike, thousand kernel weight, plant height, flag leaf area and leaf color. The association between SNP and these agronomic traits may provide new insight for study of the gene's potential contribution to drought tolerance of barley.

## Introduction

Small heat shock proteins (sHSPs) with a molecular weight of 15 to 42 kDa are produced ubiquitously in prokaryotic and eukaryotic cells under heat and other stress conditions at various growth stages [1]–[5]. Most sHSPs have strong cytoprotective effects by keeping functional conformations of proteins or refolding denatured proteins, relieving protein from aggregation and removing harmful polypeptides under stress conditions [6]. These sHSPs are classified into several subgroups based on DNA sequence similarity, immunological cross-reactivity and intracellular localization [7].

Heat shock protein 17.8 (HSP17.8), a member of class I cytosolic sHSPs, presents as a dimer under normal physiological conditions and is converted to high oligomeric complexes, ranging from 240 kDa to >480 kDa, after heat shock [3]. Several studies have postulated that *HSP17.8* plays a role in tolerance to heat [8], freezing [9], [10] and drought [11], [12]. *HSP17.8* showed chaperone-like activity to protect citrate synthase from thermal aggregation at 43°C in the cyanobacterium *Anabaena* sp. PCC 7120 [8], and played a role in membrane protein targeting to the chloroplast outer membrane in *Arabidopsis* [3]. At the freezing temperature, *HSP17.8* showed at least 1.5 times more expression in freezing-tolerant transgenic maize than in non-transgenic maize [10]. Similarly, *HSP17.8* transcripts were present at a higher level in drought-tolerant control plants of Katya cultivar than in non-tolerant Sadovo cultivar of wheat [11]. In the case of barley, *HSP17.8* showed expression exclusively in drought-tolerant barley genotypes e.g. Martin and *Hordeum spontaneum* 41-1, and not in drought-sensitive genotype e.g. Moroc9-75, under drought stress [12].

The ability of different accessions of a crop species to adapt to stress is a result of sequence variation in genes in the accessions of the given crop species. Single nucleotide polymorphisms (SNPs) and small insertions and deletions (indels) are the most common forms of genetic variation in natural populations, and is a reflection of evolution and adaptation that play a prominent role in the heritability of phenotypes [13]. EcoTILLING is the application of TILLING (Targeting Induced Local Lesions IN Genome) for discovery of SNPs and small indels in natural populations. Initially EcoTILLING was used to detect nucleotide variation in five genes in a natural population of *Arabidopsis* [14]. In the case of poplar (*Populus trichocarpa*), EcoTILLING identified 63 SNPs in nine different genes in 41 different populations [15]. In brief, several reports have indicated that EcoTILLING is an accurate, high-throughput and low-cost technique for the discovery and evaluation of nucleotide variations in candidate genes associated with target traits [16]–[18]. Recently, association analysis emerged as a powerful approach to identify the role of genetic polymorphism in phenotypic variations in response to environmental stresses [19], [20]. For instance, by using association analysis, one SNP of the *HvCBF4* gene was significantly (P<0.001) associated with salt tolerance in 188 Tibetan barley accessions [21], and five SNPs in *Lhcb1* gene were significantly (P<0.01) associated with several agronomic traits in 292 barley accessions [22]. However, no effort has been made so far to assay allelic variation in *HSP17.8* and its association with agronomic traits in barley.

In this study, EcoTILLING approach was used to detect genetic variations of *HSP17.8* in a natural barley population comprising of 210 accessions collected from diverse geographical origins. Distribution of SNPs across different geographic origins (Africa, Middle East Asia, North East Asia, Arabian Peninsula and Europe), row types (two-row or six-row) and other possible categories (such as wild versus landrace) were investigated. Population parameters were estimated using SNPs found in different barley populations. An attempt was also made to assess potential effect of SNPs on protein function and their association with six agronomic traits in barley.

## Materials and Methods

### Plant Material

Seeds of 210 barley (*Hordeum vulgare* L.) accessions containing 171 *H. vulgare* landraces and 39 wild relatives of *Hordeum vulgare* ssp. *spontaneum* (hereafter named *H. spontaneum*) were obtained from the International Centre for Agricultural Research in the Dry Areas (Table S1). Of the 210 accessions, 164 originated from 19 countries in Asia, 40 accessions from six African countries, and 6 accessions from five European countries. For the purpose of comparison, accessions were divided into five adjacent geographic regions as described in Varshney et al. [23]. The geographical distribution of investigated accessions is given in Table 1.

**Table 1:** Summary of the geographic origins of barley accessions used for allele mining of *HSP17.8*.

Geographic region	No. accessions	Countries	No. countries
Africa	40	Algeria, Egypt, Ethiopia, Libya, Morocco, Tunisia	6
North East Asia	106	Afghanistan, Azerbaijan, China, Georgia, India, Iran, Pakistan, Tajikistan, Turkey, Turkmenistan, Uzbekistan	11
Middle East Asia	44	Iraq, Jordan, Lebanon, Palestine, Syria	5
Arabian Peninsula	14	Oman, Saudi Arabia, Yemen	3
Europe	6	Albania, Bosnia and Herzegovina, Deutschland, Greece, Serbia and Montenego	5
Total	210		30

### Phenotypic data

The germplasm collection (210 accessions) was sown with three replications during two growing seasons (2009/2010 and 2010/2011) at the Experimental Station of Guangzhou University, Guangzhou, Guangdong Province, China (23° 16' N; 113°23'E, elevation 16 m asl). Eleven seeds of each accession were planted 30 cm apart in 1.5 m long, single-row plots. All accessions were evaluated for six agronomic traits—flag leaf area (FLA in cm^2^), spike length (SL in cm), number of grains per spike (NGS), leaf color (LC in SPAD), plant height (PH in cm) and thousand kernel weight (TKW in g)—in replicated field experiments. Three randomly selected plants of each accession from each plot were characterized for the six traits using the following methods:

1. Flag leaf area (FLA; cm^2^) of the uppermost, fully expanded leaf of the main tiller at flowering. FLA = leaf length×leaf width×0.75 [24].

2. Spike length (SL; cm) was measured from the base of each main spike to the top of the spike, excluding awns, at maturity.

3. Number of grains per spike (NGS) was counted at maturity and the average number of seeds from three spikes used for analysis.

4. Leaf color (LC; SPAD) was determined at heading, before any symptoms of senescence were visible. The middle section of a randomly-selected flag leaf was evaluated between 09:00 and 12:00 h using a chlorophyll meter (SPAD-502, Minolta, Japan) and averaged over two measurements for analysis.

5. Plant height (PH; cm) was measured from ground level to the base of spike at maturity.

6. Thousand kernel weight (TKW; g) was calculated based on a sample of 250 seeds per plot.

### Designing of primers

For amplification of a target fragment of *HSP17.8* as described in Wienholds et al. [25], gene-specific primers were designed according to the published mRNA sequence of *HSP17.8* from GeneBank (accession no. AK368988.1) with melting temperatures around 60°C by using Primer 5.0 software (Premier Biosoft International, Palo Alto, CA, USA) (Table S2). The region covered by the primer pair is 600 base pairs, which includes the whole open reading frame (483 bp) of *HSP17.8*. Sequence of universal primers M13 was added to 5' ends of HSPforward and HSPreverse as adaptors (Table S2). M13 forward primers labeled with IRDye800 at 5′-end and M13 reverse primers labeled with IRDye700 at 5′-end were synthesized by LI-COR Inc.

### DNA extraction and PCR

Genomic DNA of barley accessions was extracted from 200 mg young leaf tissue following Guo et al. [26]. DNA from all samples was quantified using a spectrophotometer and normalized to a concentration of 20 ng/ µl. DNA from each accession was mixed in a 1∶1 ratio with reference DNA (ICARDA IG: 26727) to generate heteroduplexes for point mutation detection.

For nucleotide polymorphism screening with EcoTILLING, the target region of *HSP17.8* was amplified by a nested PCR described by Wang et al. [27]. The first PCR was performed with a gene-specific primer pair. After the first PCR reaction, samples were diluted in 90 µl water; 1 µl diluted PCR was used as a template for the second nested PCR reaction. This reaction contained a mixture of four primers: 0.08 µM IRD800-labeled M13 forward primers, 0.02 µM M13HSPforward primers, 0.06 µM IRD700-labeled M13 reverse primers and 0.04 µM M13HSPreverse primers. After the nested PCR, heteroduplexes formation was performed by incubating the reaction mix at 99°C for 10 min, and slow renaturation with 70 cycles of 20 sec at 70°C with a decrement of 0.3°C per cycle.

**Figure 1:**
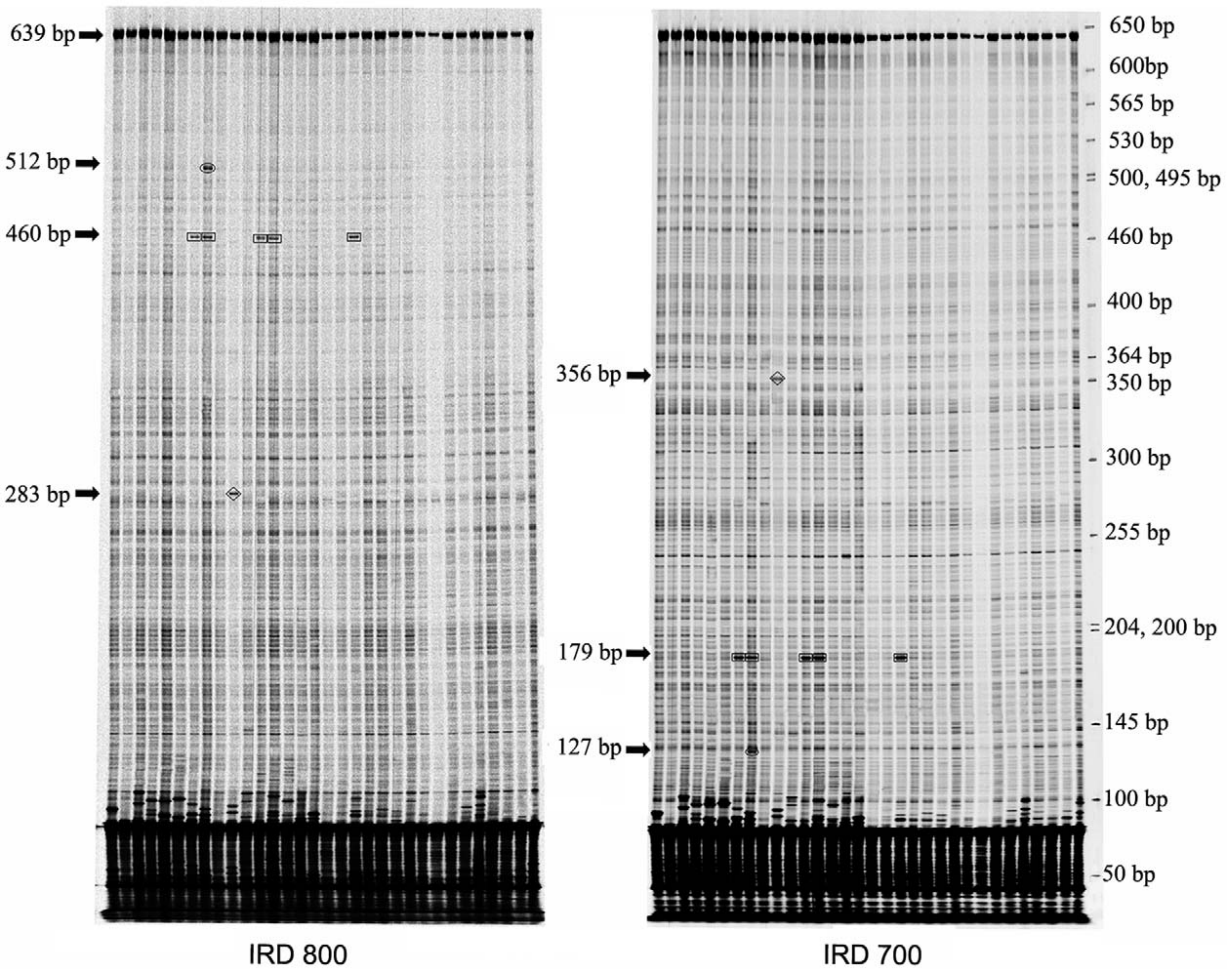
Detection of polymorphisms for a targeted region of the *HSP17.8* gene by EcoTILLING. Sampled images of the IRD 800 and IRD 700 channels are shown at left and right, respectively. The specific cleavage products appear as intense dark bands between 127 to 512 bp with molecular weights listed to the left in each channel image by arrows. Complementary fragments in corresponding lanes between the IRD 700 and IRD 800 channel images labeled with the same box pattern (including rectangle, oval and diamond). The sizes of complementary fragments in the IRD 700 labeled and the IRD 800 labeled add up to the size of PCR fragment (639 bp). Several intense dark bands near the bottom of the gel in both channels result from random mispriming. Molecular weights are provided by the GelBuddy program. The size of the DNA ladder is listed to the right of the IRD700 image.

The PCR products were digested by CEL I as described by Raghavan et al. [28]. Celery juice extract (CEL I) was produced following Guo and Li [29]. For digestion, 10 µl of solution containing 10 mM HEPES (pH 7.5), 10 mM MgSO_4_, 0.002% (w/v) Triton X-100, 0.2 µg/ml of bovine serum albumin, and 0.4 µl CEL I enzyme solution, was added to 10 µl of heteroduplex DNAs, and incubated at 45°C for 15 min. Digestion was stopped by adding 5 µl 0.25 M EDTA (pH 8.0), mixing thoroughly and then incubating on ice. Digested products were purified using isopropanol as described by Raghavan et al. [28]. After samples purification, 5 µl formamide loading dye and 10 µl ddH_2_O were added and heated at 85°C for concentrating sample to about 3 µl. Samples were loaded (0.8 µl/well) on denaturing 6.5% polyacrylamide gels on LI-COR 4300 DNA Analyzer. Two electronic image files were produced per gel run, one in the IRD700 channel and the other in IRD800 channel. Tiff images were manually scored using the GelBuddy program [30]. The appearance of cleavage products in both channels at reciprocal size that add up to the full length PCR product, were considered a polymorphic site (Fig. 1). Data summary reports generated by GelBuddy were imported to Microsoft Excel for further analysis. Samples were grouped into putative haplotype categories based on the cleaved banding pattern in evaluated gel-frames.

### DNA sequencing and statistical analysis

One representative genotype for each unique haplotype was reamplified by gene-specific primers using 40 ng of genomic DNA. The resulted PCR fragment was directly sequenced from both directions using an ABI 3730xl DNA Analyzer by a commercial company (Sangon Biotech Co., Ltd., China) to confirm the polymorphisms. Each polymorphic site was sequenced in more than one accession to confirm only two alleles segregating at any specific site. Sequences were analyzed using ClustalW software (http://www.ebi.ac.uk/tools). The SIFT (Sorting Intolerant from Tolerant) and PARSESNP (Position-Specific Scoring Matrix) programs were used to predict the impact of missense mutations on protein function [31], [32]. Population genetics parameters, including nucleotide diversity (π), haplotype diversity (HD) and Tajima's D [33], were analyzed using DnaSP v5.0 [34].

### Association analysis between SNPs and agronomic traits

Association between markers and traits was evaluated using a General Linear Model (GLM_*Q*) in the TASSEL v3.0 software (http://www.maizegenetics.net/tassel), where the SNP being tested was considered as a fixed effect, and the factor and matrix of subpopulation membership (*Q* matrix) were used as cofactors to account for population structure. Possible population structure was depicted using genotypic data of the 210 barley accessions and 21 genome-wide SSR markers (3 SSRs for each chromosome) (Table S3) by Structure software version 2 [35]. Three groups were identified (unpublished). Permutations of 1,000 runs were performed to calculate the significant *p* value for F-test. The association between a marker and a trait is represented by its R^2^ value, an estimate of the percentage of variance explained by the marker.

## Results

### Variability of phenotypic traits

Three developmental traits—flag leaf area (FLA in cm^2^), leaf color (LC in SPAD) and plant height (PH in cm) were measured for all 210 accessions (Table 2). Three yield-related traits—spike length (SL in cm), number of grains per spike (NGS) and 1000-kernel weight (TKW in g) were measured for only 192 and 195 barley accessions in the 2009/2010 and 2010/2011 growing seasons, because 18 and 15 barley accessions did not head, respectively. Large phenotypic variation was observed for all traits, and significant correlations between the various phenotypic traits were found among the barley accessions (Table 2). Two-row and 6-row barley differed significantly in all phenotypic measurements except for TKW. In addition, *H. spontaneum* accessions had significantly lower scores in FLA, NGS, LC and TKW than *H. vulgare* landraces.

**Table 2:** Phenotypic scores from field trials.

Traits a	2009/2010							2010/2011				
	SL (192)	NGS (192)	TKW (192)	LC (210)	PH (210)	FLA (210)		SL (195)	NGS (195)	TKW (195)	LC (210)	PH (210)
	cm	grains/spike	g	SPAD	cm	cm^2^		cm	grains/spike	g	SPAD	cm
Range	5.2–12.6	3.0–54.7	13.7–66.2	26.3–54.5	11.0–79.4	7.2–82.0		4.6–13.2	5.0–68.0	21.3–72.6	30.7–59.6	44.0–115.5
Average ± SD	8.5±1.4	23.6±12.2	41.5±9.7	42.4±5.9	47.6±17.1	42.6±13.5		8.5±1.7	32.9±14.7	40.6±9.0	46.6±4.8	78.2±13.3
SPON	8.4±1.7	14.2±6.5	38.5±12.2	40.2±5.4	45.1±19.2	30.0±11.4		8.7±1.8	29.1±15.7	31.0±4.4	44.5±3.8	77.5±12.6
VUL-LR	8.5±1.3	25.6±12.2**	42.2±9.0*	43.0±5.9**	48.1±16.7	45.5±12.3**		8.5±1.6	33.7±14.4	42.6±8.4**	47.1±4.9**	78.4±13.5
2-Row	8.9±1.5*	14.5±7.1	42.9±11.2	41.4±5.7	44.3±17.8	34.9±11.4		9.04±1.6**	24.2±13.4	39.4±10.6	45.9±4.5	74.5±12.8
6-Row	8.2±1.3	30.2±10.8**	40.5±8.4	43.2±5.9*	49.8±16.3*	48.1±12.2**		8.2±1.5	39.2±12.3**	41.5±7.6	47.2±5.0	80.8±13.1**
*Correlations*												
NGS	−0.019							−0.146*				
TKW	0.193**	−0.050						−0.005	−0.140			
LC	0.004	0.137	0.028					−0.171*	0.058	0.219**		
PH	−0.018	0.076	0.310**	0.259**				−0.113	1.66*	−0.086	0.049	
FLA	−0.016	0.242**	−0.077	0.211**	−0.023							

Average ± standard deviation (SD) of genotype subsets (SPON: *H. spontaneum*, VUL-LR: *H. vulgare* landraces) and different row types are given.

a FLA: flag leaf area (cm^2^), SL: spike length (cm), NGS: number of grains per spike, LC: leaf color (SPAD), PH: plant height (cm), TKW: 1000 kernel weight (g); number in bracket indicates number of plants scored and measured

* indicates significant level (*P*<0.05) of difference between means, or correlation between phenotypic traits

** indicates highly significant level (*P*<0.01) of difference between means, or correlation between phenotypic traits

### Nucleotide polymorphisms

By using EcoTILLING, 13 polymorphic sites were detected in the targeted region of *HSP17.8* across the 210 accessions (Table 3, Fig. 1). The frequency of polymorphic sites in 210 accessions ranged from 0.005 to 0.143 with an average 0.030. To determine the precise position and nature of these polymorphic sites, several samples containing each of these sites were randomly sequenced. Accessions with the same polymorphic sites in EcoTILLING exhibited the same nucleotide changes in sequence with only one exception that one sample showing two polymorphic sites in EcoTILLING did not show nucleotide variation in sequencing. Thus, sequencing confirmed 11 SNPs in the targeted region of *HSP17.8* at a frequency of 1 SNP per 54.5 bp. Among the 11 SNPs, 10 were from coding regions and one from non-coding region, and seven were transitions (C-T and A-G) and four were transversions (A-C, A-T, C-G and G-T). Among the ten coding SNPs, six were nonsynonymous mutations s and four were silent synonymous mutations. Five of the missense mutations were predicted to have a severe effect on the HSP17.8 protein function.

**Table 3:** List of nucleotide polymorphisms in *HSP17.8* and their effects on codon frequencies.

Nucleotide change^a^	Band^b^	Frequency^c^	Effect^d^	PARSESNP^e^	SIFT^f^
G104A^g^	+	0.019	Non-coding		
T204G	+	0.010	F26V	**12.3**	0.45
G267A	+	0.019	A49 =		
G300C	+	0.019	E60D	6.6	**0**
C428T	+	0.024	T103M	9.7	**0.01**
C469T	+	0.143	L117 =		
G483A	+	0.010	R121 =		
G525A	+	0.014	M135I	9.4	**0.02**
G565A	+	0.014	A149T	8.6	0.6
C582A^g^	+	0.014	I154 =		
C599G^g^	+	0.100	S160C	7.5	**0**
ND	+(407 bp)	0.005			
ND	+(394 bp)	0.005			

a First letter indicates common bp at this site, followed by position of SNP in sequence on GenBank accession number AK368988.1, and then nucleotide which is the rare variant at this site.

b All nucleotide changes identified by sequencing were first by EcoTILLING as a band on gel image. In one sample, 407 bp and 394 bp were identified on EcoTILLING gel for which a corresponding polymorphism could not be confirmed by sequencing.

c Frequency was calculated by dividing the number of similar nucleotide changes identified on EcoTILLING gel by the number of samples analyzed.

d First letter indicates the common amino acid at this site, followed by position of SNP within predicted protein sequence and then amino acid change induced by the variant nucleotide polymorphism. ‘ = ’ means no change in amino acid encoded by that codon (synonymous variation).

e A non-synonymous SNP is predicted to be damaging to encoded protein if PARSESNP score is >10 (bold).

f A non-synonymous SNP is predicted to be damaging to encoded protein if SIFT score is <0.05 (bold).

g Putative polymorphisms in gel regions with high levels of noise from primer mispriming, one fragment evidently appeared on one image channel and the corresponding fragment in alternative image channels could not be unambiguously assigned.

The nucleotide diversity in the sequenced region of *HSP17.8* as measured by π (pairwise nucleotide diversity) was 0.00188 among the 210 accessions. Across different geographic regions, the range of π values spanned from 0.00049 for African accessions (40 accessions) to 0.00212 for Middle East Asian accessions (44 accessions). Similarly, π for *H. spontaneum* was higher than for *H. vulgare* landrace accessions. In addition, 2-row and 6-row barley had similar nucleotide diversity (Table 4). To test whether the SNPs in the targeted region of *HSP17.8* were neutral mutations, Tajima's D statistics (Tajima 1989) were estimated. Tajima's D values were negative for all sub-populations except for Africa population. However none of the values was statistically significant (*P*<0.05) (Table 4), thus, nucleotide variations in *HSP17.8* gene could be the results from the standard neutral selection.

**Table 4:** Frequency of *HSP17.8* haplotypes, nucleotide diversity (π), haplotype diversity (HD) and Tajima's D test for different barley populations.

		Frequency of haplotypes^a^									No. SNPs	π	No. haplotypes	HD	Tajima's D
		H1	H2	H3	H4	H5	H6	H7	H8	H9					
Overall (210)		0.667	0.143	0.100	0.024	0.019	0.014	0.014	0.010	0.010	11	0.00118	9	0.526	−1.46671
Geographic regions^b^	AFR (40)	0.825	-	0.175	-	-	-	-	-	-	1	0.00049	2	0.296	0.37079
	MEA (44)	0.455	-	0.250	0.114	0.091	0.045	-	-	0.045	7	0.00212	6	0.722	−0.56398
	NEA (106)	0.660	0.264	0.019	-	-	0.009	0.028	0.019	-	6	0.001	6	0.497	−1.06616
	APS (14)	0.786	0.143	0.071	-	-	-	-	-	-	2	0.00068	3	0.385	−0.95919
	EUR (6)	1.000	-	-	-	-	-	-	-	-	-	-	1	-	-
Genotype subset^c^	VUL-LR (171)	0.684	0.158	0.123	-	-	0.006	0.018	0.012	-	6	0.00098	6	0.494	−0.91134
	SPON (39)	0.590	0.077	-	0.128	0.103	0.051	-	-	0.051	7	0.0019	6	0.63	−0.86422
Row type	2-row (87)	0.621	0.057	0.149	0.057	-	0.023	-	0.023	0.023	6	0.00107	7	0.55	−1.08501
	6-row (123)	0.699	0.203	0.065	-	-	0.008	0.024	-	-	5	0.00094	5	0.469	−0.81416

a Haplotypes are ordered by overall frequency in all barley accessions.

b AFR: Africa, APS: Arabian Peninsula, EUR: Europe, MEA: Middle East Asia, NEA: North East Asia.

c SPON: *H. spontaneum*; VUL-LR: *H. vulgare* landraces.

Numbers in brackets indicate number of plants scored and measured.

### Haplotype diversity analysis

For the 11 SNPs confirmed by sequencing, nine distinguishable haplotypes were detected across 210 accessions (Table 5). The level of haplotype diversity was 0.526, with the frequency of each haplotype shown in Table 4. Among all the haplotypes, three major haplotypes were detected in 210 accessions, with the cumulative frequency of the first three haplotypes being 0.9. Haplotype H1 was found in more than two-thirds of the accessions screened. Haplotype H2 was observed in 30 accessions (14.3%), and haplotype H3 observed in 21 accessions (10.0%). The frequency of the other haplotypes (H4 to H9) was low, between 1.0% and 2.4% (Table 4).

**Table 5:** Distribution of polymorphic SNPs across nine *HSP17.8* haplotypes.

Haplotypes	SNP position											Total number of accessions
	104	196	267	300	428	469	483	525	565	582	599	
H1	G	T	G	G	C	C	G	G	G	C	C	140
H2	G	T	G	G	C	**T**	G	G	G	C	C	30
H3	G	T	G	G	C	C	G	G	G	C	**G**	21
H4	G	T	G	G	**T**	C	G	G	G	C	C	5
H5	**A**	T	**A**	**C**	C	C	G	G	G	C	C	4
H6	G	T	G	G	C	C	G	G	G	**A**	C	3
H7	G	T	G	G	C	C	G	**A**	**A**	C	C	3
H8	G	T	G	G	C	C	**A**	G	G	C	C	2
H9	G	**G**	G	G	C	C	G	G	G	C	C	2

SNPs relative to the most common sequence (haplotype H1) are indicated in bold. The number of SNP positions is relative to the sequence on GenBank accession number AK368988.1

**Figure 2:**
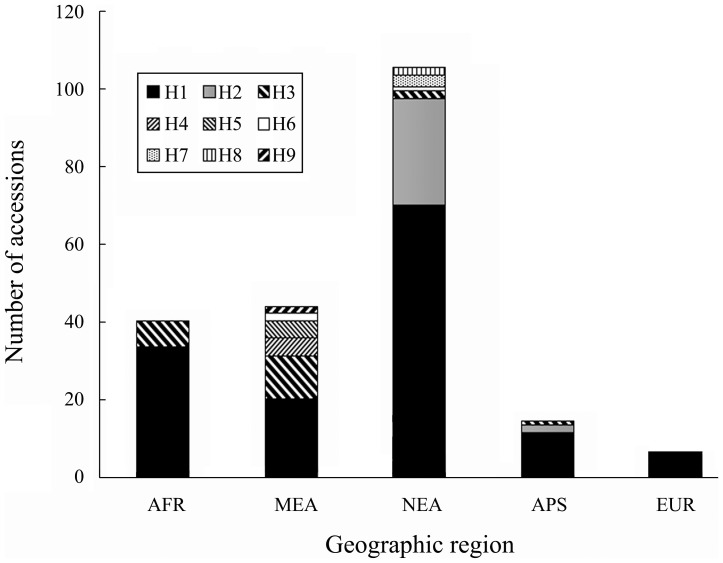
Composition of *HSP17.8* haplotype in accessions of different geographic region. AFR: Africa, APS: Arabian Peninsula, EUR: Europe, MEA: Middle East Asia, NEA: North East Asia. H (H1–H9) represents haplotype as described in Table 5.

The frequencies of *HSP17.8* haplotypes differed markedly across geographical regions (Table 4 and Fig. 2). Haplotype diversity ranged from 0 in Europe (6 accessions) to 0.722 in the Middle East Asia (44 accessions). This was particularly evident for the haplotype H2 which was absent in Africa, Middle East Asia and Europe, but most frequent in North East Asia (0.264). The less frequently identified haplotypes were confined to specific geographic regions. Of the six haplotypes present in <10% of accessions sampled, five were unique to one region (Asia); three of which were exclusive to accessions from Middle East Asia; and two from North East Asia. In addition, *H. spontaneum* and *H. vulgare* landraces were widely separated into six haplotypes with only three common between the two groups (Table 4). When comparing 2-row and 6-row barley, more haplotypes and higher diversity were observed in 2-row barley (Table 4).

### Association between SNPs and phenotypic traits

Because two SNPs (positions 196 bp and 483 bp in *HSP17.8*) were rare alleles (frequency <1%), and three SNPs (positions 104 bp, 267 bp and 300 bp) or two SNPs (positions 525 bp and 565 bp) were in complete linkage disequilibrium, five SNPs (positions 196 bp, 483 bp, 104 bp, 267 bp and 565 bp) were excluded from further analysis. Although two SNPs (position 469 and 582 bp) were synonymous variations, they may be in complete linkage disequilibrium with other non-synonymous mutations, which could result in biological changes in the organism. Therefore, these two SNPs were further analyzed. Thus only six SNPs (positions 300 bp, 428 bp, 469 bp, 525 bp, 582 bp and 599 bp) were used in association analysis. Significant association (*P*<0.05) was observed for four distinct SNPs with at least one of the evaluated traits except for spike length, and four of the associations were highly significant (*P*<0.01) (Table 6). These associated SNPs explained 2.0% to 4.6% of the variation for individual traits. FLA was significantly associated (*P*<0.01) with one SNP (position 469 bp in *HSP17.8*) in 2009/2010, which explained 3.9% of the phenotypic variation. NGS was significantly associated (*P*<0.01) with one SNP at position 599 bp, which explained 3.3% and 4.6% phenotypic variation in 2009/2010 and 2010/2011, respectively. One SNP (position 300 in *HSP17.8*) significantly associated (*P*<0.05) with PH was observed, accounting for approximately 2% phenotypic variation in 2009/2010 and 2010/2011. LC showed significant (*P*<0.05) association with three SNPs (position 469 bp, 525 bp and 599 bp in *HSP17.8*), which explained 2.8%, 3.9% and 2.4% of the phenotypic variation, respectively. TGW was significantly associated (*P*<0.05) with one SNP at position 300 bp in 2009/2010, which explained 2.6% phenotypic variation.

**Table 6:** SNPs of *HSP17.8* associated with agronomic traits of barley using a significance level corresponding to α = 0.05.

Growing season	Traits	SNP position	F	P	R^2^	Elite allele	Frequency of elite allele
2009/2010	FLA	469 C>T	10.44**	0.0014	0.039	T	16.67%
	NGS	599 C>G	7.07**	0.0085	0.033	C	89.06%
	LC	469 C>T	6.13*	0.0141	0.028	T	16.67%
	TGW	300 G>C	5.78*	0.0172^a^	0.026	G	98.10%
	PH	300 G>C	5.08*	0.0252^a^	0.023	G	98.10%
2010/2011	LC	525 G>A	10.26**	0.0016^a^	0.039	G	98.57%
	NGS	599 C>G	9.80**	0.0020	0.046	C	89.23%
	LC	599 C>G	6.09*	0.0145	0.024	C	90.00%
	PH	300 G>C	4.42*	0.0368^a^	0.020	G	98.10%

FLA, flag leaf area (cm^2^); NGS, number of grains per spike; LC, leaf color (SPAD); PH, plant height (cm); TKW, thousand kernel weight (g).

Number of SNP positions is relative to the sequence on GenBank accession number AK368988.1

R^2^ is the fraction of total variation explained by the marker.

a Due to low minor allele frequency, these results should be evaluated with caution.

* (*P*<0.05) indicates SNP significantly associated with traits.

** (*P*<0.01) indicates SNP highly significantly associated with traits.

## Discussion

### Natural variation in the barley population

To characterize genetic variation in *HSP17.8*, 171 *H. vulgare* landraces and 39 *H. spontaneum* accessions from 30 countries in three continents were characterized for allele diversity. EcoTILLING revealed 11 SNPs and nine haplotypes after analysis of 126,000 bp sequences in *HSP17.8*. Of the 11 unique SNPs, ten were in the coding region, which included four silent synonymous mutations and six missense mutations. Of the six missense mutations, four SNPs were predicted by the SIFT program to severely affect protein function, and one mutation was predicted by PSSM program to severely damage the function of the predicted protein. Because there is a 20% false-positive error in SIFT [31], some mutations predicted to be deleterious may be functionally neutral. However, these scores may be useful in prioritizing mutations for further study and analysis of possible contributions and roles of *HSP17.8* in stress tolerance.

Tajima's D is used to measure deviation from neutral evolution by comparing diversity estimates based on nucleotide diversity (θ) and average pairwise nucleotide diversity (π). Parameters θ and π are affected differently by natural selection. In this study, Tajima's D neutrality test revealed no evidence of natural selection for *HSP17.8* (Table 4) but under some kind of purifying selection as revealed by a high negative value of Tajima's D. This insignificant result may be attributed to the low number of SNPs observed, which weakens the neutrality test. This result agrees with previous reports on other functional genes in barley [36] and *CPsHSP-2* in *Machilus kusano* [37]. However, the Tajima's D value in Africa was positive, which may result from balancing selection or bottleneck effect.

Compared to previous reports on barley [36], [38], nucleotide diversity (π = 0.00118) of *HSP17.8* was lower, and the level of haplotype diversity (0.526) was higher in this study. The average frequency of SNPs was 1 per 54.5 bp, which was similar to that found in Chen et al. (1 SNP/53.8 bp) [39], lower than Zeng et al.(1 SNP/9.8 bp) [40], and higher than Rostoks et al. (1 SNP/200 bp) [41]. The discrepancy in SNP frequency among studies may be due to differences in genomic regions assayed, and number, content and geographic origins of germplasm used [38], [42], [43]. As predicted, *H. spontaneum* had a higher nucleotide diversity (π) and haplotype diversity than *H. vulgare* landraces in this study, which agrees with previous reports [38], [44], [45]. It is likely that *H. vulgare* landraces would have gone through a population bottleneck during domestication, which resulted in a reduction in genetic diversity[38], [45]. Comparing genetic diversity across geographical regions, accessions from Middle East Asia exhibited the highest nucleotide diversity (π), which agrees with studies of Malysheva-Otto et al. [46] and Varshney et al. [23]. One plausible reason was that the Middle East's ‘fertile crescent’ region was the center of origin of barley and the main distribution region of wild barley [47], [48]. This important result may indicate that variations in *HSP17.8* in wild barleys and Middle East landraces might offer elite alleles for the improvement of stress tolerance in barley. To date, a number of wild barley and Middle East types have been used in barley breeding programs [49].

Different ecotypes of barley germplasm show their specific traits in terms of ecology, morphology and physiology [50]. Different ecotype populations from several different geographic regions may include abundant genetic variation associated with phenotypic traits. As extremely low nucleotide diversity could be observed in one ecotype, only a small number of ecotype populations may be sufficient to capture already adequate genetic variability [51]. Since wild and cultivated barley are cross-compatible, it is possible to increase the genetic diversity of barley using wild ancestors as a parent in crosses with cultivated barley [52]. There is evidence that the introgression of chromosome segments from wild progenitors improves agronomic performance of an elite cultivar [52]–[54].

### Association analysis between SNPs and phenotypic traits

In our previous studies, *HSP17.8* in five barley accessions, namely Martin, HS4-1, Moroc9-75, Tadmor and WI2291, was up-regulated under drought stress with the expression fold changes of 15.67, 19.43, 23.92, 2.48 and 1.15, respectively [12], [55]. Simple linear regression analysis between expression fold changes and six agronomic traits was performed in five barley accessions, and significant correlations were observed for PH, NGS, TKW, FLA and LC with the R^2^ values of 0.138, 0.163, 0.169, 0.134 and 0.218, respectively, suggesting the high expression level of *HSP17.8* is likely associated with PH, NGS, TKW, FLA and LC. In addition, BLAST search in GenBank (http://www.ncbi.nlm.nih.gov/genbank/) showed that *HSP17.8* exhibited nucleotide sequence homology to a barley EST (GenBank accession AL510041) that was located in 63.4 cM on chromosome 4H [56]. Several previous studies also reported chromosome 4H had QTLs for PH [57]–[59], TGW [57]–[59], NGS [58]–[60], LC [61] and FLA [62]. Therefore, *HSP17.8* may have cis affects for the QTLs of PH, TGW, NGS, LC, and FLA located on chromosome 4H.

Association mapping has recently emerged as an alternative approach to mapping QTL and genes associated with quantitative traits using a diverse collection of germplasm lines or breeding materials [63]. Compared to traditional QTL mapping, association mapping is faster and provides greater capacity and power for QTL/gene detection [19], [20]. In addition, candidate-gene association analysis is more precise than genome-wide association analysis. Because association analysis links specific nucleotide polymorphisms to trait variations, one may be able to associate SNPs with specific biological effects [64]. Many agronomically important traits are controlled by QTLs [65]. By association analysis of 816 genome-wide markers, Varshney et al. [66] identified one to eight significant QTLs for nine agronomic traits in 223 barley accessions. With 204 polymorphisms in 24 transcription families, Yu et al. [67] found three genes associated with the drought tolerance index and five genes with the drought tolerance level in 95 diverse rice landraces. In the present study, four SNPs in the *HSP17.8* gene were significantly associated with at least one agronomic trait investigated. Three SNPs at positions 469 bp, 525 bp and 599 bp of *HSP17.8* were significantly associated with LC, one SNP at position 469 bp with FLA, one SNP at position 599 bp with NGS, and one SNP at position 300 bp with PH and TGW. Of the four SNPs associated with phenotypic traits, three at positions 300 bp, 525 bp and 599 bp in *HSP17.8* were missense mutations which, according to SIFT, were predicted to severely affect protein function. Association analysis showed that the three missense mutations were deleterious mutations. Due to low minor allele frequency for two SNPs (positions 300 and 525), their association results should be interpreted with caution. However, one SNP at position 469 associated with phenotypic traits was a synonymous mutation that may not change the structure of the gene product. A plausible reason for this result is the hitchhiking effort of a locus in positive selection [68] or a false positive association. Thus, these SNPs identified by association analysis need to be validated for individual cultivars involved in crosses before they can be applied to marker-assisted selection in the progeny [69], [70]. The findings from this study indicate that further research of these newly detected SNPs in *HSP17.8* is necessary to evaluate their possible influence on agronomic traits and usefulness as future selection markers in modern barley breeding program.

## Conclusions

In this study, 11 SNPs were detected and nine unique haplotypes were identified in *HSP17.8* among 210 barley accessions collected from 30 countries by EcoTILLING technology, and 5 SNPs with missense changes are predicted to be deleterious to protein function. Four SNPs significantly associated with agronomic traits identified in this study can be used as DNA markers in marker-assisted selection to improve these agronomic traits after further validation of their functions in individual cultivars.

## Supporting Information

Table S1 **Details on barley accessions used in this study.**
(DOC)

Table S2 **Primer sequences used for PCR amplification of** ***HSP17.8*** **.**
(DOC)

Table S3 **SSR marker information used in evaluation of population structure.**
(DOC)
