# Supplementary material for: Single Nucleotide Polymorphisms in HSP17.8 and Their Association with Agronomic Traits in Barley
Source: PLoS One. 2013 Feb 13;8(2):e56816. doi: 10.1371/journal.pone.0056816 (PMC3572059; doi:10.1371/journal.pone.0056816)
Supplement: Table S1 — Details on barley accessions used in this study. (DOC) [file pone.0056816.s001.doc]

**Table S1 Details on barley accessions used in this study**

| **ICARDA IG** | **Origin country^a^** | **Donor country** | **Genotype description (species)** | **Ear type** |
| --- | --- | --- | --- | --- |
| 16981 | TKM | USA | *Hordeum vulgare* subsp. vulgare convar. vulgare | 6-row |
| 17424 | SCG | USA | *Hordeum vulgare* subsp. vulgare convar. vulgare | 6-row |
| 18767 | TUR | USA | *Hordeum vulgare* subsp. vulgare convar. vulgare | 6-row |
| 18983 | GRC | USA | *Hordeum vulgare* subsp. vulgare convar. vulgare | 6-row |
| 19390 | CHN | USA | *Hordeum vulgare* subsp. vulgare convar. vulgare | 6-row |
| 19391 | CHN | USA | *Hordeum vulgare* subsp. vulgare convar. vulgare | 6-row |
| 19453 | IRN | USA | *Hordeum vulgare* subsp. vulgare convar. vulgare | 6-row |
| 19687 | CHN | USA | *Hordeum vulgare* subsp. vulgare convar. vulgare | 6-row |
| 20905 | AFG | USA | *Hordeum vulgare* subsp. vulgare convar. vulgare | 6-row |
| 23248 | ETH | USA | *Hordeum vulgare* subsp. vulgare convar. vulgare | 6-row |
| 23515 | ETH | USA | *Hordeum vulgare* subsp. vulgare convar. vulgare | 6-row |
| 24634 | DZA | USA | *Hordeum vulgare* subsp. vulgare convar. vulgare | 6-row |
| 24745 | AZE | USA | *Hordeum vulgare* subsp. vulgare convar. vulgare | 6-row |
| 24746 | TKM | USA | *Hordeum vulgare* subsp. vulgare convar. vulgare | 6-row |
| 24751 | CHN | USA | *Hordeum vulgare* subsp. vulgare convar. vulgare | 6-row |
| 24764 | CHN | USA | *Hordeum vulgare* subsp. vulgare convar. vulgare | 6-row |
| 24774 | CHN | USA | *Hordeum vulgare* subsp. vulgare convar. vulgare | 6-row |
| 24944 | CHN | USA | *Hordeum vulgare* subsp. vulgare convar. vulgare | 6-row |
| 24953 | LBY | USA | *Hordeum vulgare* subsp. vulgare convar. vulgare | 6-row |
| 25704 | IND | USA | *Hordeum vulgare* subsp. vulgare convar. vulgare | 6-row |
| 25839 | EGY | USA | *Hordeum vulgare* subsp. vulgare convar. vulgare | 6-row |
| 25883 | AFG | USA | *Hordeum vulgare* subsp. vulgare convar. vulgare | 6-row |
| 26002 | AZE | USA | *Hordeum vulgare* subsp. vulgare convar. vulgare | 6-row |
| 26055 | CHN | USA | *Hordeum vulgare* subsp. vulgare convar. vulgare | 6-row |
| 26056 | CHN | USA | *Hordeum vulgare* subsp. vulgare convar. vulgare | 6-row |
| 26081 | CHN | USA | *Hordeum vulgare* subsp. vulgare convar. vulgare | 6-row |
| 26172 | CHN | USA | *Hordeum vulgare* subsp. vulgare convar. vulgare | 6-row |
| 26178 | CHN | USA | *Hordeum vulgare* subsp. vulgare convar. vulgare | 6-row |
| 26229 | CHN | USA | *Hordeum vulgare* subsp. vulgare convar. vulgare | 6-row |
| 26276 | IRQ | USA | *Hordeum vulgare* subsp. vulgare convar. vulgare | 6-row |
| 26727 | AZE | USA | *Hordeum vulgare* subsp. vulgare convar. vulgare | 6-row |
| 27076 | CHN | USA | *Hordeum vulgare* subsp. vulgare convar. vulgare | 6-row |
| 27630 | PAK | USA | *Hordeum vulgare* subsp. vulgare convar. vulgare | 6-row |
| 27649 | IRN | USA | *Hordeum vulgare* subsp. vulgare convar. vulgare | 6-row |
| 27653 | IRN | USA | *Hordeum vulgare* subsp. vulgare convar. vulgare | 6-row |
| 27683 | AFG | USA | *Hordeum vulgare* subsp. vulgare convar. vulgare | 6-row |
| 27773 | IRN | USA | *Hordeum vulgare* subsp. vulgare convar. vulgare | 6-row |
| 27790 | IRN | USA | *Hordeum vulgare* subsp. vulgare convar. vulgare | 6-row |
| 27794 | IRN | USA | *Hordeum vulgare* subsp. vulgare convar. vulgare | 6-row |
| 27799 | AFG | USA | *Hordeum vulgare* subsp. vulgare convar. vulgare | 6-row |
| 27803 | AFG | USA | *Hordeum vulgare* subsp. vulgare convar. vulgare | 6-row |
| 29097 | JOR | SYR | *Hordeum vulgare* subsp. vulgare convar. vulgare | 6-row |
| 31406 | SYR | SYR | *Hordeum vulgare* subsp. vulgare convar. vulgare | 6-row |
| 31410 | SYR | SYR | *Hordeum vulgare* subsp. vulgare convar. vulgare | 6-row |
| 31870 | MAR | MAR | *Hordeum vulgare* subsp. vulgare convar. vulgare | 6-row |
| 31876 | MAR | MAR | *Hordeum vulgare* subsp. vulgare convar. vulgare | 6-row |
| 31923 | MAR | MAR | *Hordeum vulgare* subsp. vulgare convar. vulgare | 6-row |
| 31938 | MAR | MAR | *Hordeum vulgare* subsp. vulgare convar. vulgare | 6-row |
| 32475 | EGY | SYR | *Hordeum vulgare* subsp. vulgare convar. vulgare | 6-row |
| 32482 | EGY | SYR | *Hordeum vulgare* subsp. vulgare convar. vulgare | 6-row |
| 32488 | EGY | SYR | *Hordeum vulgare* subsp. vulgare convar. vulgare | 6-row |
| 32601 | PAK | SYR | *Hordeum vulgare* subsp. vulgare convar. vulgare | 6-row |
| 32608 | PAK | SYR | *Hordeum vulgare* subsp. vulgare convar. vulgare | 6-row |
| 32618 | PAK | SYR | *Hordeum vulgare* subsp. vulgare convar. vulgare | 6-row |
| 32708 | SYR | SYR | *Hordeum vulgare* subsp. vulgare convar. vulgare | 6-row |
| 32711 | SYR | SYR | *Hordeum vulgare* subsp. vulgare convar. vulgare | 6-row |
| 32826 | OMN | ITA | *Hordeum vulgare* subsp. vulgare convar. vulgare | 6-row |
| 32954 | OMN | GBR | *Hordeum vulgare* subsp. vulgare convar. vulgare | 6-row |
| 32962 | OMN | ITA | *Hordeum vulgare* subsp. vulgare convar. vulgare | 6-row |
| 32971 | OMN | ITA | *Hordeum vulgare* subsp. vulgare convar. vulgare | 6-row |
| 32977 | OMN | ITA | *Hordeum vulgare* subsp. vulgare convar. vulgare | 6-row |
| 33024 | DZA | SYR | *Hordeum vulgare* subsp. vulgare convar. vulgare | 6-row |
| 33055 | DZA | SYR | *Hordeum vulgare* subsp. vulgare convar. vulgare | 6-row |
| 33102 | DZA | SYR | *Hordeum vulgare* subsp. vulgare convar. vulgare | 6-row |
| 33195 | CHN | CHN | *Hordeum vulgare* subsp. vulgare convar. vulgare | 6-row |
| 33608 | CHN | CHN | *Hordeum vulgare* subsp. vulgare convar. vulgare | 6-row |
| 33649 | CHN | CHN | *Hordeum vulgare* subsp. vulgare convar. vulgare | 6-row |
| 34263 | CHN | CHN | *Hordeum vulgare* subsp. vulgare convar. vulgare | 6-row |
| 35382 | DZA | SYR | *Hordeum vulgare* subsp. vulgare convar. vulgare | 6-row |
| 35385 | DZA | SYR | *Hordeum vulgare* subsp. vulgare convar. vulgare | 6-row |
| 35386 | DZA | SYR | *Hordeum vulgare* subsp. vulgare convar. vulgare | 6-row |
| 35792 | CHN | CAN | *Hordeum vulgare* subsp. vulgare convar. vulgare | 6-row |
| 35794 | CHN | CAN | *Hordeum vulgare* subsp. vulgare convar. vulgare | 6-row |
| 35800 | CHN | CAN | *Hordeum vulgare* subsp. vulgare convar. vulgare | 6-row |
| 35803 | CHN | CAN | *Hordeum vulgare* subsp. vulgare convar. vulgare | 6-row |
| 35806 | CHN | CAN | *Hordeum vulgare* subsp. vulgare convar. vulgare | 6-row |
| 35808 | CHN | CAN | *Hordeum vulgare* subsp. vulgare convar. vulgare | 6-row |
| 35814 | CHN | CAN | *Hordeum vulgare* subsp. vulgare convar. vulgare | 6-row |
| 35820 | CHN | CAN | *Hordeum vulgare* subsp. vulgare convar. vulgare | 6-row |
| 35822 | CHN | CAN | *Hordeum vulgare* subsp. vulgare convar. vulgare | 6-row |
| 35823 | CHN | CAN | *Hordeum vulgare* subsp. vulgare convar. vulgare | 2-row |
| 35826 | CHN | CAN | *Hordeum vulgare* subsp. vulgare convar. vulgare | 6-row |
| 36052 | LBY | SYR | *Hordeum vulgare* subsp. vulgare convar. vulgare | 6-row |
| 37525 | PAK | USA | *Hordeum vulgare* subsp. vulgare convar. vulgare | 6-row |
| 37554 | LBY | ITA | *Hordeum vulgare* subsp. vulgare convar. vulgare | 6-row |
| 37556 | LBY | ITA | *Hordeum vulgare* subsp. vulgare convar. vulgare | 6-row |
| 37576 | LBY | ITA | *Hordeum vulgare* subsp. vulgare convar. vulgare | 6-row |
| 37726 | TUN | TUN | *Hordeum vulgare* subsp. vulgare convar. vulgare | 6-row |
| 37729 | TUN | TUN | *Hordeum vulgare* subsp. vulgare convar. vulgare | 6-row |
| 37784 | TUN | TUN | *Hordeum vulgare* subsp. vulgare convar. vulgare | 6-row |
| 37813 | TUN | TUN | *Hordeum vulgare* subsp. vulgare convar. vulgare | 6-row |
| 38214 | CHN | CAN | *Hordeum vulgare* subsp. vulgare convar. vulgare | 6-row |
| 107010 | IRN | SYR | *Hordeum vulgare* subsp. vulgare convar. vulgare | 6-row |
| 107020 | IRN | SYR | *Hordeum vulgare* subsp. vulgare convar. vulgare | 6-row |
| 108499 | PAK | SYR | *Hordeum vulgare* subsp. vulgare convar. vulgare | 6-row |
| 108911 | IRQ | IRQ | *Hordeum vulgare* subsp. vulgare convar. vulgare | 6-row |
| 112483 | GEO | DEU | *Hordeum vulgare* subsp. vulgare convar. vulgare | 6-row |
| 112715 | IRN | DEU | *Hordeum vulgare* subsp. vulgare convar. vulgare | 6-row |
| 112840 | LBY | DEU | *Hordeum vulgare* subsp. vulgare convar. vulgare | 6-row |
| 112865 | LBY | DEU | *Hordeum vulgare* subsp. vulgare convar. vulgare | 6-row |
| 112931 | TUR | DEU | *Hordeum vulgare* subsp. vulgare convar. vulgare | 6-row |
| 113084 | SAU | ITA | *Hordeum vulgare* subsp. vulgare convar. vulgare | 2-row |
| 113120 | IRN | IRN | *Hordeum vulgare* subsp. vulgare convar. vulgare | 6-row |
| 113126 | IRN | IRN | *Hordeum vulgare* subsp. vulgare convar. vulgare | 6-row |
| 113128 | IRN | IRN | *Hordeum vulgare* subsp. vulgare convar. vulgare | 6-row |
| 115919 | LBY | DEU | *Hordeum vulgare* subsp. vulgare convar. vulgare | 6-row |
| 120565 | TKM | RUS | *Hordeum vulgare* subsp. vulgare convar. vulgare | 6-row |
| 123901 | UZB | SYR | *Hordeum vulgare* subsp. vulgare convar. vulgare | 6-row |
| 123923 | UZB | SYR | *Hordeum vulgare* subsp. vulgare convar. vulgare | 6-row |
| 125827 | AZE | RUS | *Hordeum vulgare* subsp. vulgare convar. vulgare | 6-row |
| 128122 | IRN | SYR | *Hordeum vulgare* subsp. vulgare convar. vulgare | 6-row |
| 128124 | IRN | SYR | *Hordeum vulgare* subsp. vulgare convar. vulgare | 6-row |
| 128125 | IRN | SYR | *Hordeum vulgare* subsp. vulgare convar. vulgare | 6-row |
| 128133 | IRN | SYR | *Hordeum vulgare* subsp. vulgare convar. vulgare | 6-row |
| 128158 | PAK | SYR | *Hordeum vulgare* subsp. vulgare convar. vulgare | 6-row |
| 128159 | PAK | SYR | *Hordeum vulgare* subsp. vulgare convar. vulgare | 6-row |
| 128160 | PAK | SYR | *Hordeum vulgare* subsp. vulgare convar. vulgare | 6-row |
| 128170 | DZA | SYR | *Hordeum vulgare* subsp. vulgare convar. vulgare | 6-row |
| 128187 | EGY | SYR | *Hordeum vulgare* subsp. vulgare convar. vulgare | 6-row |
| 128199 | JOR | SYR | *Hordeum vulgare* subsp. vulgare convar. vulgare | 6-row |
| 128204 | EGY | SYR | *Hordeum vulgare* subsp. vulgare convar. vulgare | 6-row |
| 128218 | LBY | SYR | *Hordeum vulgare* subsp. vulgare convar. vulgare | 6-row |
| 135258 | JOR | SYR | *Hordeum vulgare* subsp. vulgare convar. vulgare | 6-row |
| 135528 | TKM | SYR | *Hordeum vulgare* subsp. vulgare convar. vulgare | 6-row |
| 137761 | TJK | SYR | *Hordeum vulgare* subsp. vulgare convar. vulgare | 6-row |
| 22957 | ETH | USA | *Hordeum vulgare* subsp. vulgare convar. distichon | 2-row |
| 27892 | SAU | USA | *Hordeum vulgare* subsp. vulgare convar. distichon | 2-row |
| 17406 | BIH | USA | *Hordeum vulgare* subsp. vulgare convar. vulgare | 2-row |
| 17410 | BIH | USA | *Hordeum vulgare* subsp. vulgare convar. vulgare | 2-row |
| 19620 | ALB | USA | *Hordeum vulgare* subsp. vulgare convar. vulgare | 2-row |
| 20900 | AFG | USA | *Hordeum vulgare* subsp. vulgare convar. vulgare | 2-row |
| 22912 | ETH | USA | *Hordeum vulgare* subsp. vulgare convar. vulgare | 2-row |
| 24720 | TKM | USA | *Hordeum vulgare* subsp. vulgare convar. vulgare | 2-row |
| 25095 | CHN | USA | *Hordeum vulgare* subsp. vulgare convar. vulgare | 2-row |
| 25327 | CHN | USA | *Hordeum vulgare* subsp. vulgare convar. vulgare | 2-row |
| 25710 | IND | USA | *Hordeum vulgare* subsp. vulgare convar. vulgare | 2-row |
| 25843 | EGY | USA | *Hordeum vulgare* subsp. vulgare convar. vulgare | 2-row |
| 25947 | CHN | USA | *Hordeum vulgare* subsp. vulgare convar. vulgare | 2-row |
| 25961 | CHN | USA | *Hordeum vulgare* subsp. vulgare convar. vulgare | 2-row |
| 27655 | IRN | USA | *Hordeum vulgare* subsp. vulgare convar. vulgare | 2-row |
| 27784 | IRN | USA | *Hordeum vulgare* subsp. vulgare convar. vulgare | 2-row |
| 28674 | TUR | USA | *Hordeum vulgare* subsp. vulgare convar. vulgare | 2-row |
| 28677 | TUR | USA | *Hordeum vulgare* subsp. vulgare convar. vulgare | 2-row |
| 28693 | TUR | USA | *Hordeum vulgare* subsp. vulgare convar. vulgare | 2-row |
| 28865 | DEU | USA | *Hordeum vulgare* subsp. vulgare convar. vulgare | 2-row |
| 29057 | SYR | SYR | *Hordeum vulgare* subsp. vulgare convar. vulgare | 2-row |
| 31396 | SYR | SYR | *Hordeum vulgare* subsp. vulgare convar. vulgare | 2-row |
| 31412 | SYR | SYR | *Hordeum vulgare* subsp. vulgare convar. vulgare | 2-row |
| 32687 | EGY | SYR | *Hordeum vulgare* subsp. vulgare convar. vulgare | 2-row |
| 32694 | EGY | SYR | *Hordeum vulgare* subsp. vulgare convar. vulgare | 2-row |
| 32756 | SYR | SYR | *Hordeum vulgare* subsp. vulgare convar. vulgare | 2-row |
| 32774 | SYR | SYR | *Hordeum vulgare* subsp. vulgare convar. vulgare | 2-row |
| 32812 | EGY | SYR | *Hordeum vulgare* subsp. vulgare convar. vulgare | 2-row |
| 32814 | OMN | ITA | *Hordeum vulgare* subsp. vulgare convar. vulgare | 2-row |
| 32978 | OMN | ITA | *Hordeum vulgare* subsp. vulgare convar. vulgare | 2-row |
| 33094 | SYR | SYR | *Hordeum vulgare* subsp. vulgare convar. vulgare | 2-row |
| 35220 | SYR | SYR | *Hordeum vulgare* subsp. vulgare convar. vulgare | 2-row |
| 35236 | SYR | SYR | *Hordeum vulgare* subsp. vulgare convar. vulgare | 2-row |
| 36058 | TJK | SYR | *Hordeum vulgare* subsp. vulgare convar. vulgare | 2-row |
| 37608 | YEM | ITA | *Hordeum vulgare* subsp. vulgare convar. vulgare | 2-row |
| 37612 | YEM | ITA | *Hordeum vulgare* subsp. vulgare convar. vulgare | 2-row |
| 112781 | IRN | DEU | *Hordeum vulgare* subsp. vulgare convar. vulgare | 2-row |
| 113076 | SAU | ITA | *Hordeum vulgare* subsp. vulgare convar. vulgare | 2-row |
| 113082 | SAU | ITA | *Hordeum vulgare* subsp. vulgare convar. vulgare | 2-row |
| 113095 | YEM | ITA | *Hordeum vulgare* subsp. vulgare convar. vulgare | 2-row |
| 128088 | AFG | SYR | *Hordeum vulgare* subsp. vulgare convar. vulgare | 2-row |
| 128172 | SYR | SYR | *Hordeum vulgare* subsp. vulgare convar. vulgare | 2-row |
| 128173 | SYR | SYR | *Hordeum vulgare* subsp. vulgare convar. vulgare | 2-row |
| 128200 | JOR | SYR | *Hordeum vulgare* subsp. vulgare convar. vulgare | 2-row |
| 128202 | JOR | SYR | *Hordeum vulgare* subsp. vulgare convar. vulgare | 2-row |
| 131668 | TJK | SYR | *Hordeum vulgare* subsp. vulgare convar. vulgare | 2-row |
| 38215 | CHN | CAN | *Hordeum vulgare* subsp. spontaneum | 2-row |
| 38611 | SYR | SYR | *Hordeum vulgare* subsp. spontaneum | 2-row |
| 38638 | SYR | ITA | *Hordeum vulgare* subsp. spontaneum | 2-row |
| 38660 | AFG | USA | *Hordeum vulgare* subsp. spontaneum | 2-row |
| 38669 | AFG | USA | *Hordeum vulgare* subsp. spontaneum | 2-row |
| 38672 | TUR | USA | *Hordeum vulgare* subsp. spontaneum | 2-row |
| 38693 | PAK | SYR | *Hordeum vulgare* subsp. spontaneum | 2-row |
| 38956 | PAL | USA | *Hordeum vulgare* subsp. spontaneum | 2-row |
| 39126 | PAL | USA | *Hordeum vulgare* subsp. spontaneum | 2-row |
| 39540 | LBN | USA | *Hordeum vulgare* subsp. spontaneum | 2-row |
| 39802 | PAK | SWE | *Hordeum vulgare* subsp. spontaneum | 2-row |
| 39847 | SYR | SYR | *Hordeum vulgare* subsp. spontaneum | 2-row |
| 39857 | SYR | SYR | *Hordeum vulgare* subsp. spontaneum | 2-row |
| 39891 | EGY | SYR | *Hordeum vulgare* subsp. spontaneum | 2-row |
| 40019 | JOR | SYR | *Hordeum vulgare* subsp. spontaneum | 2-row |
| 40021 | JOR | SYR | *Hordeum vulgare* subsp. spontaneum | 2-row |
| 40022 | JOR | SYR | *Hordeum vulgare* subsp. spontaneum | 2-row |
| 40031 | JOR | SYR | *Hordeum vulgare* subsp. spontaneum | 2-row |
| 40034 | JOR | SYR | *Hordeum vulgare* subsp. spontaneum | 2-row |
| 40035 | JOR | SYR | *Hordeum vulgare* subsp. spontaneum | 2-row |
| 40039 | JOR | SYR | *Hordeum vulgare* subsp. spontaneum | 2-row |
| 40056 | JOR | SYR | *Hordeum vulgare* subsp. spontaneum | 2-row |
| 40059 | JOR | SYR | *Hordeum vulgare* subsp. spontaneum | 2-row |
| 40064 | JOR | SYR | *Hordeum vulgare* subsp. spontaneum | 2-row |
| 40071 | JOR | SYR | *Hordeum vulgare* subsp. spontaneum | 2-row |
| 40072 | JOR | SYR | *Hordeum vulgare* subsp. spontaneum | 2-row |
| 40082 | SYR | SYR | *Hordeum vulgare* subsp. spontaneum | 2-row |
| 40101 | TKM | SYR | *Hordeum vulgare* subsp. spontaneum | 2-row |
| 40104 | TKM | SYR | *Hordeum vulgare* subsp. spontaneum | 2-row |
| 107046 | IRN | SYR | *Hordeum vulgare* subsp. spontaneum | 2-row |
| 107427 | IRQ | SYR | *Hordeum vulgare* subsp. spontaneum | 2-row |
| 110742 | SYR | SYR | *Hordeum vulgare* subsp. spontaneum | 2-row |
| 112787 | IRN | DEU | *Hordeum vulgare* subsp. spontaneum | 2-row |
| 115781 | JOR | SYR | *Hordeum vulgare* subsp. spontaneum | 2-row |
| 120794 | TKM | JPN | *Hordeum vulgare* subsp. spontaneum | 2-row |
| 132606 | AZE | SYR | *Hordeum vulgare* subsp. spontaneum | 2-row |
| 135507 | TKM | SYR | *Hordeum vulgare* subsp. spontaneum | 2-row |
| 135536 | TKM | SYR | *Hordeum vulgare* subsp. spontaneum | 2-row |
| 135624 | TKM | SYR | *Hordeum vulgare* subsp. spontaneum | 2-row |

^a^Standard code for country of origin, e.g. ALB=Albania, AFG=Afghanistan, AZE=Azerbaijan, BIH=Bosnia and Herzegovina, CHN=China, DEU=Deutschland, DZA= Algeria, EGY= Egypt, ETH= Ethiopia, GEO=Georgia, GRC=Greece, IND= India, IRN= Iran, IRQ= Iraq, JOR= Jordan, LBN= Lebanon, LBY= Libya, MAR= Morocco, OMN= Oman, PAK=Pakistan, PAL= Palestine, RUS= Russia, SAU=Saudi Arabia, SYR= Syria, SCG=Serbia and Montenego, TJK= Tajikistan, TKM=Turkmenistan, TUN= Tunis, TUR=Turkey, UZB= Uzbekistan, YEM=Yemen
