# Supplementary material for: Single Nucleotide Polymorphisms in HSP17.8 and Their Association with Agronomic Traits in Barley
Source: PLoS One. 2013 Feb 13;8(2):e56816. doi: 10.1371/journal.pone.0056816 (PMC3572059; doi:10.1371/journal.pone.0056816)
Supplement: Table S2 — Primer sequences used for PCR amplification of HSP17.8 . (DOC) [file pone.0056816.s002.doc]

**Table S2 Primer sequences used for PCR amplification of *HSP17.8***

| Primer name | Sequence 5′ → 3′ |
| --- | --- |
| HSPforward | GCTTATAACACATCCGAAGC |
| HSPreverse | CAGCGAACTGAACCTTAATC |
| M13HSPforward | cacgacgttgtaaaacgacGCTTATAACACATCCGAAGC |
| M13HSPreverse | ggataacaatttcacacaggCAGCGAACTGAACCTTAATC |
| M13-F (IRDye800) | cacgacgttgtaaaacgac |
| M13-R (IRDye700) | ggataacaatttcacacagg |
