# Supplementary material for: Single Nucleotide Polymorphisms in HSP17.8 and Their Association with Agronomic Traits in Barley
Source: PLoS One. 2013 Feb 13;8(2):e56816. doi: 10.1371/journal.pone.0056816 (PMC3572059; doi:10.1371/journal.pone.0056816)
Supplement: Table S3 — SSR marker information used in evaluation of population structure. (DOC) [file pone.0056816.s003.doc]

**Table S3** **SSR marker information used in evaluation of population structure**

| Chromosome | Marker name | Forward primer sequence (5'→3') | Revers primer sequence (5'→3') |
| --- | --- | --- | --- |
| 1H | Bmac90 | ACATCAACCCTCCTGCTC | CCGCACATAGTGGTTACATC |
| 1H | Bmag105 | AATCAGACCCATCAGAGGT | CCGGTCTCATAGAAATGG |
| 1H | HVALAAT | TACATACAACCCTCATGGG | AAGGATGACATGGCTTTG |
| 2H | Bmag125 | AATTAGCGAGAACAAAATCAC | AGATAACGATGCACCACC |
| 2H | Ebmac415 | GAAACCCATCATAGCAGC | AAACAGCAGCAAGAGGAG |
| 2H | HVBKASI | ATTGGCGTGACCGATATTTATGTTCA | CAAAACTGCAGCTAAGCAGGGGAACA |
| 3H | Bmag13 | AAGGGGAATCAAAATGGGAG | TCGAATAGGTCTCCGAAGAA |
| 3H | HVLTPPB | AGACGCTGAGTACGTTGAG | CAAAGTACAACAAACTCACGA |
| 3H | Hvm33 | ATATTAAAAAAGGTGGAAAGCC | CACGCCCTCTCCCTAGAT |
| 4H | Bmac30 | CCCAATCGGAGTTACAGATG | GCCTCTCTGAGAATGGATC |
| 4H | Gms89 | TGAAGTGGAAGGCTTCGC | GCTCTCGTTGTGCGGAG |
| 4H | HVMLOH1A | CCTCCCCTCTGATATGATAA | GTACAGACGGTTTAATTGTCC |
| 5H | Bmag5 | TCCATGATGATGTGTGCATAGA | CGGATCCCAACAAACACAC |
| 5H | Gms1 | CTGACCCTTTGCTTAACATGC | TCAGCGTGACAAACAATAAAGG |
| 5H | HVLOX | CAGCATATCCATCTGATCTG | CACCCTTATTTATTGCCTTAA |
| 6H | Bmac316 | ATGGTAGAGGTCCCAACTG | ATCACTGCTGTGCCTAGC |
| 6H | Hvm74 | AGGAAGTCATTGCGTGAG | TGATCAAGAATGATAACATGG |
| 6H | Scssr5599 | TTCCATCATAACAGCAATGG | TTCGTCGAAGGCTATGTAGG |
| 7H | Bmac156 | AACCGAATGTATTCCTCTGTA | GCCAAACAACTATCGTGAC |
| 7H | Bmag11 | ACAAAAACACCGCAAAGAAGA | GCTAGTACCTAGATGACCCCC |
| 7H | HVAMY2 | CTGTAAGTGAGACAATCGACA | CAGTTGAACCCCTGAAAG |

Primer sequences and locations for SSRs used in this study were obtained from the GrainGenes database (<http://www.wheat.pw.usda.gov/> GG2/index.shtml).
